# Dual-task interference as a function of varying motor and cognitive demands

**DOI:** 10.3389/fpsyg.2022.952245

**Published:** 2022-09-29

**Authors:** Anna Michelle McPhee, Theodore C. K. Cheung, Mark A. Schmuckler

**Affiliations:** Department of Psychology, University of Toronto Scarborough, Toronto, ON, Canada

**Keywords:** dual-task performance, locomotion, counting behavior, kinematic measures, dual-task costs

## Abstract

Multitasking is a critical feature of our daily lives. Using a dual-task paradigm, this experiment explored adults’ abilities to simultaneously engage in everyday motor and cognitive activities, counting while walking, under conditions varying the difficulty of each of these tasks. Motor difficulty was manipulated by having participants walk forward versus backward, and cognitive difficulty was manipulated by having participants count forward versus backward, employing either a serial 2 s or serial 3 s task. All of these manipulations were performed in single-task conditions (walk only, count only) and dual-task conditions (walk and count simultaneously). Both motor performance variables (cycle time, stride length, walking velocity) and cognitive variables (counting fluency, counting accuracy) were assessed in these conditions. Analyses of single-task conditions revealed that both motor and cognitive manipulations predictably influenced performance. Analyses of dual-task performance revealed influences of motor and cognitive factors on both motor and cognitive performance. Most centrally, dual-task costs (normalized difference between single- and dual-task conditions) for motor variables revealed that such costs occurred primarily for temporal or spatiotemporal gait parameters (cycle time, walking velocity) and were driven by cognitive manipulations. Dual-task cost analyses for cognitive measures revealed negative dual-task costs, or dual-task benefits, for cognitive performance. Finally, the effects of dual-task manipulations were correlated for motor and cognitive measures, indicating dual-task performance as a significant individual difference variable. These findings are discussed with reference to theories of attentional allocation, as well as the possible role of auditory–motor entrainment in dual-task conditions.

## Introduction

In the world in which we live, multitasking is increasingly a part of our daily lives. People send emails and texts while walking along, and unfortunately crossing the street, have business meetings while driving in their cars, or even simply carry on unrelated conversations while preparing and cooking a complex meal. Activities such as these require that people split their attention while performing multiple simultaneous behaviors. Technically, the idea of multitasking in this fashion represents what has been called “dual-task performance,” and researchers across a range of disciplines and expertise have been interested in exploring this varied and complex behavior. According to Koch and colleagues, investigations of “multitasking can reveal fundamental aspects of cognitive architecture and the mechanisms of information processing” (Koch et al., 2018, p: 557).

The study of dual-task behavior has grown substantially over the decades (see McIsaac et al., 2015, for a review), with such investigations ranging from classic work on divided attention (Kahneman, 1973; Norman and Bobrow, 1975; Navon and Gopher, 1979) to the impact of concurrent cognitive processing and motor performance in healthy young and older adults (Huang and Mercer, 2001; Woollacott and Shumway-Cooke, 2002; Al-Yahya et al., 2011; Leone et al., 2017) and children (Huang and Mercer, 2001; Hinton and Vallis, 2015, 2016), and within clinical settings (Plummer et al., 2013; Schott et al., 2016; Learmonth et al., 2017; He et al., 2018; Schott and Klotzbier, 2018; Leone et al., 2020). Within such investigations, the action of multitasking or dual-task behavior can focus on tasks that are sequential, involving the ability to shift back and forth between these behaviors, or on tasks that are simultaneous, requiring the ability to monitor and engage continuously in two divergent activities. Psychologically, the first of these frameworks involves questions of representations and/or one’s cognitive flexibility in navigating these shifts. The second of these frameworks involves the processing of simultaneous streams of information, integrating concurrent performance, or both.

Regardless of which of these frameworks is employed, the principal issue explored in dual-task settings typically involves the limitations produced in such contexts, focusing on the inherent costs of performing multiple sequential or simultaneous behaviors. Ultimately, the question examined is how does one task suffer because of the engagement in another task? Within such a broad framework, one critical dimension on which one can categorize dual-task research involves the dimensions chosen for study, along with how these dimensions are manipulated and combined. Looking across dual-task research, the majority of this work manipulates the cognitive dimension of the concurrent tasks, while leaving the motor component constant (Huang et al., 2003; Montero-Odasso et al., 2012; Katz-Leurer et al., 2014; Nascimbeni et al., 2015; Yang et al., 2017; Möhring et al., 2021), although there are notable exceptions in which the motor component is also manipulated (Schulz et al., 2010; Beurskens and Bock, 2013; Mazaheri et al., 2014, 2015; Worden and Vallis, 2014; Beurskens et al., 2016).

Studies that have focused on manipulating the cognitive dimension have typically employed tasks that vary the cognitive faculties involved, so as to examine which cognitive ability or abilities are principally involved in interacting with the motor component of the task. As an example, Patel et al. (2014) purposefully chose distinctly varying cognitive tasks, including visuomotor reaction time, word list generation, a Stroop task, and a serial subtraction task, for their investigation, based on the idea that these tasks targeted different cognitive functions. These authors found significant differences in the dual-task costs for motor performance as a function of the type of cognitive task, with relatively low costs for a visuomotor reaction time task, but a high cost for a Stroop task. Similarly, Huang et al. (2003) employed visual identification, auditory identification, and memorization tasks while walking, with the expectation that tasks relying on the use of visual processing would interfere more than other tasks because of the importance of visual information in locomotion. Interestingly, these authors observed the greatest dual-task costs on motor behavior for auditory identification, and the lowest costs for memorization, although these effects did vary as a function of gait parameters (e.g., cadence versus step length). Overall, multiple studies fall into this pattern of employing cognitive tasks varying in their underlying processes (Katz-Leurer et al., 2011, 2014; Montero-Odasso et al., 2012; Nascimbeni et al., 2015; Schott and Klotzbier, 2018; Möhring et al., 2021). Such research has highlighted that different cognitive faculties have differential influences on dual-task behavior.

As mentioned previously, researchers have also manipulated (albeit less frequently) the motor component of the dual-task paradigm (Schulz et al., 2010; Beurskens and Bock, 2013; Mazaheri et al., 2014, 2015; Worden and Vallis, 2014; Beurskens et al., 2016), to explore the impact of such variation on cognitive processing. Theoretically, manipulating the motor component is important in that common motor behaviors such as reaching for objects, maintaining balance, and walking, are typically assumed to proceed automatically. As taken axiomatically in the field, automatic behaviors are seen as occurring without the need for any cognitive resources or monitoring (i.e., Wheatley and Wegner, 2001). Thus, manipulation of such presumed automatic behavior should not impact cognitive processing in any form.

Somewhat in contrast to the cognitive manipulations just discussed, one important dimension of motor manipulations has involved varying the difficulty level of the motor task (e.g., Beurskens and Bock, 2013; Mazaheri et al., 2014, 2015), as opposed to simply varying the types of motor behavior (e.g., one-legged standing, 10-meter walking) required (although see Schulz et al., 2010; Hinton and Vallis, 2015, 2016; Beurskens et al., 2016 for examples of this latter form of manipulation). For example, Beurskens and Bock (2013) had younger and older adult participants walk along a pathway in four different conditions – a wide path at a preferred pace, a narrow path at a preferred pace, a wide path at a fast pace, and a wide path with obstacles at a preferred pace. Walking occurred simultaneously with either a visual processing task or a fine motor task. Examination of dual-task costs (the normalized difference between dual- and single-task performance) for participants’ gait parameters in these conditions revealed that these costs increased with age, with this effect significantly greater with the visual task, relative to the fine motor task. Most interestingly, this effect was modulated by the difficulty of the walking task, with the elderly participants most effected by different walking conditions (a narrow path and avoiding obstacles). As a second example, Broeker et al. (2021) varied the predictability of the components of a dual-task consisting of a continuous tracking task and an auditory–motor task, providing either advanced information in the tracking task and for the sound sequences. Participants increased performance for the predictable tasks, but not for the unpredictable tasks, suggesting a lack of increased resource investment in the latter context. Thus, motor components have also been manipulated in investigations of resources brought to bear in dual-task contexts.

One consistent theme running throughout the majority of this work is that the variation employed in the dual tasks, be they cognitive or motor, presumably modulate the difficulty of the underlying tasks. Thus, the different conditions are typically meant to represent increasing challenges to actors, thereby increasing the resources required for the experimental tasks. Indeed, the idea that resources devoted to one component of a dual-task will detract from performance in the other component is fundamental to one of the principal accounts of dual-task effects – central resource theory (Kahneman, 1973; Neumann, 1996; Wickens, 2008, 2021). With respect to the manipulation of task difficulty, the assumption is that such variation makes even greater demands on this single resource, resulting in more significant decrements in the performance of the concurrent component. A more detailed discussion of central resource accounts and the alternative theory of multiple resource accounts (Navon and Gopher, 1979; Wickens, 2002, 2008, 2021) is deferred to the discussion.

One limitation inherent to this idea, however, is that this purported difficulty variation is by and large simply implied or assumed by the researchers. As such, it is not guaranteed that the variation in the tasks employed truly represents differing “levels” of cognitive and/or motoric challenge, or if they do, what the degree of increased difficulty such changes represent. Although these manipulations typically do appear to intuitively access varying levels of difficulty, such as serial subtraction by 2 s versus 7 s, or having participants walk down wide versus narrow pathways, this idea of consistently employing manipulations that explicitly vary difficulty is not commonplace. Given this inconsistency, when assessing the impact of dual-task conditions to single-task conditions (i.e., dual-task costs), it is difficult to dissociate the impact of single-task behavior on dual-task performance *per se* (i.e., simply having to do two things) from the relative difficulty of the dual tasks. Accordingly, one goal of the current work was to compare dual-task performance across more systematically varied levels of difficulty, with performance in single-task conditions, to assess the impact of varying levels of difficulty on cognitive-motor resource requirements on behavior.

A second characteristic of previous work on dual-task behavior is that researchers, again by and large, typically manipulate only one dimension in their investigations. Thus, the impact of modifying cognitive tasks is explored while maintaining only a single motor task, or the motor requirements are modified while maintaining consistent cognitive demands. Although potentially insightful, such work misses the opportunity to examine concurrent manipulations of cognitive and motor contexts within a common set of participants and experimental contexts. The current project addresses this limitation by exploring the simultaneous manipulation of both cognitive and motor components, within the context of varying levels of difficulty, as described earlier.

Finally, a third characteristic of the previous literature, and one likely stemming from the previous limitation, is that the dual-task costs assessed generally focus on only a single dimension, such as exploring the dual-task costs on cognitive performance or motor behavior exclusively. Although some investigators do calculate and examine dual-task costs in *both* cognitive and motor dimensions simultaneously (e.g., Patel et al., 2014; Langhanns and Müller, 2018), it is rare that investigators explicitly compare and correlate dual-task costs in both cognitive and motor behavior. Accordingly, an additional goal of the current work is to relate the degree of dual-task costs in motor behavior and cognitive performance on an individual level. Put more simply, are dual-task costs for motor and cognitive behavior correlated within individuals, or do these costs vary independently? If, in fact, dual-task performance across motor and cognitive domains are related within individuals, this raises the intriguing possibility that susceptibility to such influences might represent some form of individual difference variable. Although such a claim would indeed be bold and require significant data collection across a large participant pool for validation and acceptance, the current study provides an initial exploration of this idea by assessing the relation between dual-task motor and cognitive performance on an individual participant basis.

These goals were addressed by having young adults walk under conditions of varying cognitive and motor difficulty. Motor manipulations involved forward versus backward walking, with backward walking generally considered more difficult than forward walking (Nadeau et al., 2003; Laufer, 2005; Mazaheri et al., 2015; Walsh and Taylor, 2019), often producing less complex walking as indicated by multiple gait parameters (Laufer, 2005). Cognitive manipulations involved counting forward versus backward, with this counting employing either serial 2 s or 3 s. These cognitive manipulations thus provide varying levels of difficulty depending on both the direction of the count (addition is less difficult than subtraction, Eifermann and Etzion, 1964) and the number by which individuals counted (serial 2 s versus 3 s). Both motor and cognitive behavior were compared to baseline values to determine the degree of dual-task interference experienced.

## Materials and methods

### Participants

Thirty undergraduate students from the University of Toronto Scarborough either volunteered their time, or received course credit in Introductory Psychology, for participating. Data from four of these participants were excluded due to technological errors, and data from two additional participants were removed due to experimenter error when conducting this study. Accordingly, the final sample of participants included twenty-four undergraduates (*M* = 19.42 years old; *SD* = 1.25 years; *Range* 18–22 years), ^1^none of whom reported a history of developmental delay or significant consequences arising from a severe head injury (i.e., a concussion).^2^ The study was approved by the ethical review board of the university (ethics protocol # 22699).

### Experimental apparatus

Motor behavior was assessed by analyzing kinematic gait parameters, collected using a GAITRite^®^ Electronic Walkway Plus system (v. 485, CIR Systems, Inc., 2014). The GAITRite system involves an electronic mat with an active area of 16 feet (4.88 m) long by 2 feet (0.61 m) wide. This mat consists of 18,432 pressure sensors positioned in a 348 × 48 sensor grid, producing a spatial resolution of 0.5 inches (1.27 cm), with these sensors sampled at 120 Hz. This system provides an array of spatial, temporal, and spatiotemporal kinematic variables, and has proven to be a valid and reliable measure of participants’ gait (McDonough et al., 2001; Webster et al., 2005; Van Bloemendaal et al., 2019).

### Experimental conditions

This study manipulated motor behavior by varying *Walking Direction*, with participants required to perform forward and backward walking along the pathway. As already discussed, forward versus backward walking varies walking difficulty (Nadeau et al., 2003; Laufer, 2005; Mazaheri et al., 2015; Walsh and Taylor, 2019). Thus, backward walking modifies multiple gait parameters, including decreasing walking velocity, stride length, and swing phase, and increasing cycle time, double support phase, and so on.

This study manipulated cognitive behavior by having participants perform a mental arithmetic task, with this task varying along two dimensions. The first dimension involved the *Counting Direction* of the task, with participants counting forward (addition) or backward (subtraction). As highlighted earlier, subtraction tasks are generally more challenging for participants (Eifermann and Etzion, 1964), as indicated by work on indirect addition (Torbeyns et al., 2009, 2011; Verschaffel et al., 2021). The second dimension manipulated the *Counting Difficulty* of the task by having participants employ either serial 2 s (count by 2 s) or serial 3 s (count by 3 s) tasks. Although difficulty in such tasks is typically accomplished using a serial 3 s versus serial 7 s tasks (see Bristow et al., 2016, for a review), pilot work revealed the serial 7 s task to be especially devastating to performance, with the serials 3 s task in and of itself presenting a significant cognitive challenge compared to serials 2 s. Accordingly, we modified this task to employ serial 2 s versus serial 3 s to vary the level of difficulty. All participants received a random starting number for counting tasks (between 21 and 70 for counting forward, and 49 and 98 for counting backward).

Ultimately, this study fully crossed the *Walking Direction* (walk forward, walk backward), *Counting Difficulty* (serial 2 s, serial 3 s), and Counting *Direction* (count forward, count backward) manipulations, producing 8 dual-task conditions in all. Participants also completed 6 single-task conditions in which each of these manipulations occurred in isolation. For single-task cognitive trials, counting occurred over a 10-s interval. These 14 conditions (8 dual-task and 6 single-task conditions) were run in a blocked fashion, with each block consisting of four repetitions of each trial type. The order of the experimental blocks was randomized for all participants.

### Experimental procedure

All participants provided written informed consent before taking part in the study. Participants were told that they were taking part in a study on multitasking, and that they would be asked to perform a series of motor and cognitive tasks. The various single-task and dual-task conditions were then explained to participants. In the dual-task conditions, participants were told that they should consider the motor task as the primary task, and the cognitive task as the secondary task. The choice of prioritization of tasks was primarily pragmatic (to ensure participant safety while walking, particularly in backward walking conditions). Interestingly, recent previous work on prioritization (e.g., Cnossen et al., 2004; Levy and Pashler, 2008; Jansen et al., 2016; Beurskens and Muehlbauer, 2020; Plummer et al., 2020; Wrightson et al., 2020) suggests that the impact of prioritization on walking or cognitive tasks varies with multiple factors, including the physical and cognitive abilities of the walker, and the physical and cognitive demands of the task itself. For instance, Wrightson et al. (2020) found that prioritization of a walking task reduced the dual-task impact of cognitive behavior across varying walking conditions, whereas prioritization of a cognitive task reduced the dual-task impact on walking parameters only during overground walking, relative to treadmill walking.

Participants removed their shoes and socks, and then began a short set of practice trials to familiarize them with forward and backward walking in the lab, after which they began the experimental trials. To control for accelerations and decelerations of participants’ gait as they began and ended each trial (see Abbruzzese et al., 2014), participants began at a “Start” line located 6.6 feet (2.01 m) before the GAITRite mat, and walked to the “Finish” line located 6.6 feet after the mat. After completing the trials, participants were informed as to the purposes of the experiment. The experimental protocol itself lasted approximately 30 min, with the entire visit to the lab lasting 45 min.

### Data preprocessing

#### Motor performance

Walking trials were preprocessed to ensure that the GAITRite system was able to recognize footfalls during the traversal along the pathway. For a small number of trials in a handful of participants, the system was unable to unambiguously determine footfalls; accordingly, these trials were removed from analysis. Thus, these participants had three, as opposed to four, repetitions of trials in these conditions.

Although the GAITRite system provides multiple kinematic variables of motor performance, this work focused on a single, representative measure of temporal, spatial, and spatiotemporal gait characteristics, respectively. For temporal parameters, analyses focused on *cycle time*, which is the time (in seconds) between the first contact of consecutive footfalls of the same leg and foot. For spatial parameters, analyses focused on *stride length*, which is the straight line distance (in cm) between the heel contact of consecutive footfalls of the same leg/foot. For spatiotemporal parameters, analyses focused on *walking velocity*, which involves dividing the total distance traveled by the total time taken to walk that distance.

#### Cognitive performance

Participants were instructed to count continuously from the given starting number, regardless of whether or not an error occurred. Thus, if participants were counting forward by 2 s and made an error such as, “24, 26, 29…” participants were asked to continue counting from the error (i.e., from the number “29”), meaning that the subsequent sequence of numbers would be “31, 33, 35….”

Two measures of cognitive performance were derived from records of these sessions. The first measure involved *counting fluency*, which was the number of digits per second spoken during single-task and dual-task trials. For single-task conditions, counting fluency was calculated over a 10-s time window. For dual-task conditions, counting fluency involved the number of digits recited, while participants traversed the mat (ambulation time). In both cases, counting fluency was thus the number of digits recited divided by the total time available to recite digits, producing a standardized measure of digits/s. The second measure involved *counting accuracy*, which was the number of errors made during the counting task. Again, this measure was standardized by dividing these values by the time available to say digits, and thus to make errors.

Analyses of motor (kinematic variables) and cognitive (counting measures) data focused on comparing performance in the single-task and dual-task conditions, as well as examining dual-task costs. Dual-task costs reflect the impact of performing simultaneous motor and cognitive tasks, relative to performing only a single task, and are calculated by taking the difference between single-task and dual-task conditions, divided by the single-task condition, multiplied by 100 to create percentages (Schwenk et al., 2010; Zhou et al., 2014; Manor et al., 2016). This formula indicates the impact on behavior of performing two tasks, relative to one task, normalized by performance of the single task. Typically, dual-task costs are presented as negative values to indicate their decrement in performance. Analysis of single-task conditions tests whether the experimental manipulations do, in fact, modulate motor and cognitive performance in an expected fashion. Analyses of the dual-task conditions and the dual-task costs assess the impact of performing two tasks, as opposed to one task, as a function of the various experimental conditions.

## Results

### Data analysis

#### Motor performance

**Figure 1:**
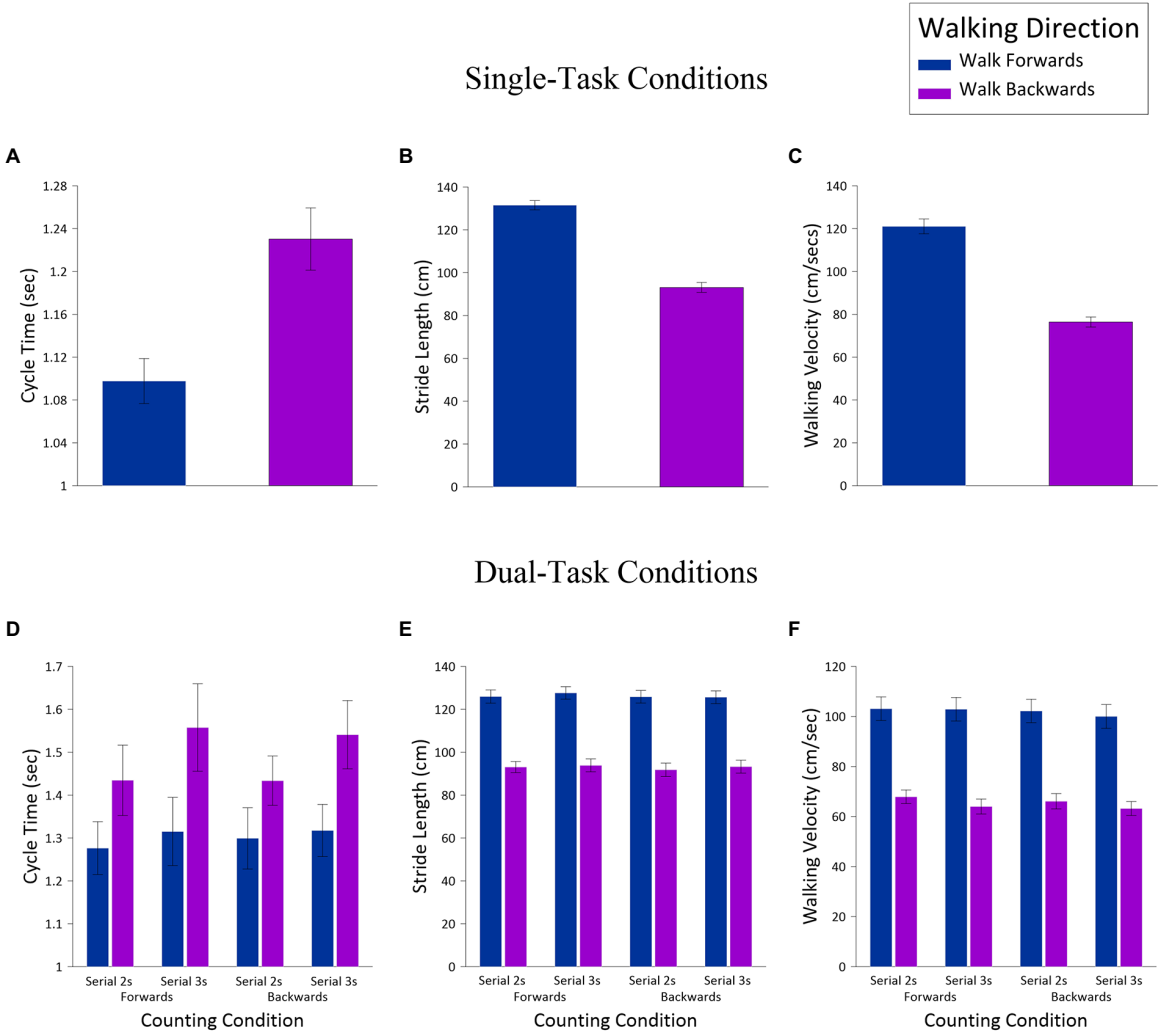
Single-task motor performance (kinematic) measures [cycle time (**A**), stride length (**B**), and walking velocity (**C**) and dual-task measures cycle time (**D**), stride length (**E**), and walking velocity (**F**)], as a function *Walking Direction* (walk forward, walk backward), *Counting Direction* (count forward, count backward), and *Counting Difficulty* (serial 2 s, serial 3 s).

Kinematic variables in the single-task and dual-task conditions were initially analyzed separately in a series of t-tests and *Analyses of Variance* (ANOVAs); Figure 1 presents data for both single- and dual-task conditions. For the single-task conditions, cycle time (Figure 1A), stride length (Figure 1B), and walking velocity (Figure 1C) were all analyzed in a series of paired t-tests, with the factor of *Walking Direction* (walk forward, walk backward). The results of these analyses can be summarized succinctly: For all three variables, walking direction significantly influenced motor performance in a predictable fashion. Thus, relative to walking forward, walking backward induced longer cycle times, *t*(23) = 6.90, *p* < 0.001, shorter stride lengths, *t*(23) = 17.07, *p* < 0.001, and slower velocities, *t*(23) = 16.15, *p* < 0.001. All of these differences are a direct consequence of the increased motor demands of backward, versus forward, walking.

The dual-task conditions were analyzed in three-way ANOVAs, with the within-subjects factors of *Walking Direction* (walk forward, walk backward), *Counting Direction* (count forward, count backward), and *Counting Difficulty* (serial 2 s, serial 3 s); Figures 1D–F shows these data as a function of these three factors. For cycle time (Figure 1D), there were significant main effects for *Walking Direction*, *F*(1,23) = 33.89, *MSE* = 0.05, *p* < 0.001, *np^2^* = 0.60, and *Counting Difficulty*, *F*(1,23) = 9.70, *MSE* = 0.03, *p* = 0.005, *np^2^* = 0.30, but no effect for *Counting Direction*, *F*(1,23) = 0.01, *MSE* = 0.03, *ns*. For *Walking Direction*, this difference indicated faster cycle times when walking forward (*M* = 1.30, *SE* = 0.07) relative to walking backward (*M* = 1.47, *SE* = 0.08), and for *Counting Difficulty*, there were faster cycle times for serial 2 s (*M* = 1.36, *SE* = 0.06) than serial 3 s (*M* = 1.43, *SE* = 0.08). The only other significant effect was the interaction between *Walking Direction* and *Counting Difficulty*, *F*(1,23) = 4.75, *MSE* = 0.02, *p* = 0.04, *np^2^* = 0.17. This interaction revealed faster cycle times for walking forward relative to walking backward for serial 2 s (*Ms* = 1.29 and 1.43, *SEs* = 0.07 and 0.07, respectively), *t*(23) = 4.26, *p* <. 001, and serial 3 s (*Ms* = 1.32 and 1.55, *SEs* = 0.07 and 0.09, respectively), *t*(23) = 5.59, *p* <. 001. However, when walking forward, there was no difference between serial 2 s and 3 s, *t*(23) = 1.29, *ns*, whereas when walking backward, there was a difference as a function of counting difficulty, *t*(23) = 3.11, *p* = 0.005.

For stride length (Figure 1E), the only significant result was a main effect for *Walking Direction*, *F*(1,23) = 222.23, *MSE* = 239.14, *p* < 0.001, *np^2^* = 0.91, with forward walking leading to longer strides (*M* = 126.3, *SE* = 2.89) than backward walking (*M* = 93.0, *SE* = 2.77). That said, the main effect of *Counting Direction* approached significance, *F*(1,23) = 3.57, *MSE* = 13.17, *p* = 0.07, *np^2^* = 0.13, with a trend for serial 2 s to produce longer stride lengths than serial 3 s (*Ms* = 110.13 and 109.14, *SEs* = 2.60 and 2.63, respectively).

Finally, for walking velocity (Figure 1F) there were significant main effects for *Walking Direction*, *F*(1,23) = 168.72, *MSE* = 382.34, *p* < 0.001, *np^2^* = 0.88 and for *Counting Difficulty*, *F*(1,23) = 6.46, *MSE* = 40.59, *p* = 0.02, *np^2^* = 0.22. The former effect revealed faster velocities when walking forward (*M* = 102.06, *SE* = 4.59) relative to backward (*M* = 65.29, *SE* = 2.68), and the latter effect revealed faster velocities for serial 2 s (*M* = 84.85, *SE* = 3.50) than serial 3 s (*M* = 82.50, *SE* = 3.52). Although the main effect for *Counting Direction* did not reach statistical significance, *F*(1,23) = 3.26, *MSE* = 39.45, *p* = 0.08, *np^2^* = 0.12, there was a noteworthy trend toward faster velocities when counting forward (*M* = 84.49, *SE* = 3.55) than counting backward (*M* = 82.85, *SE* = 3.48). None of the remaining effects were significant.

**Figure 2:**
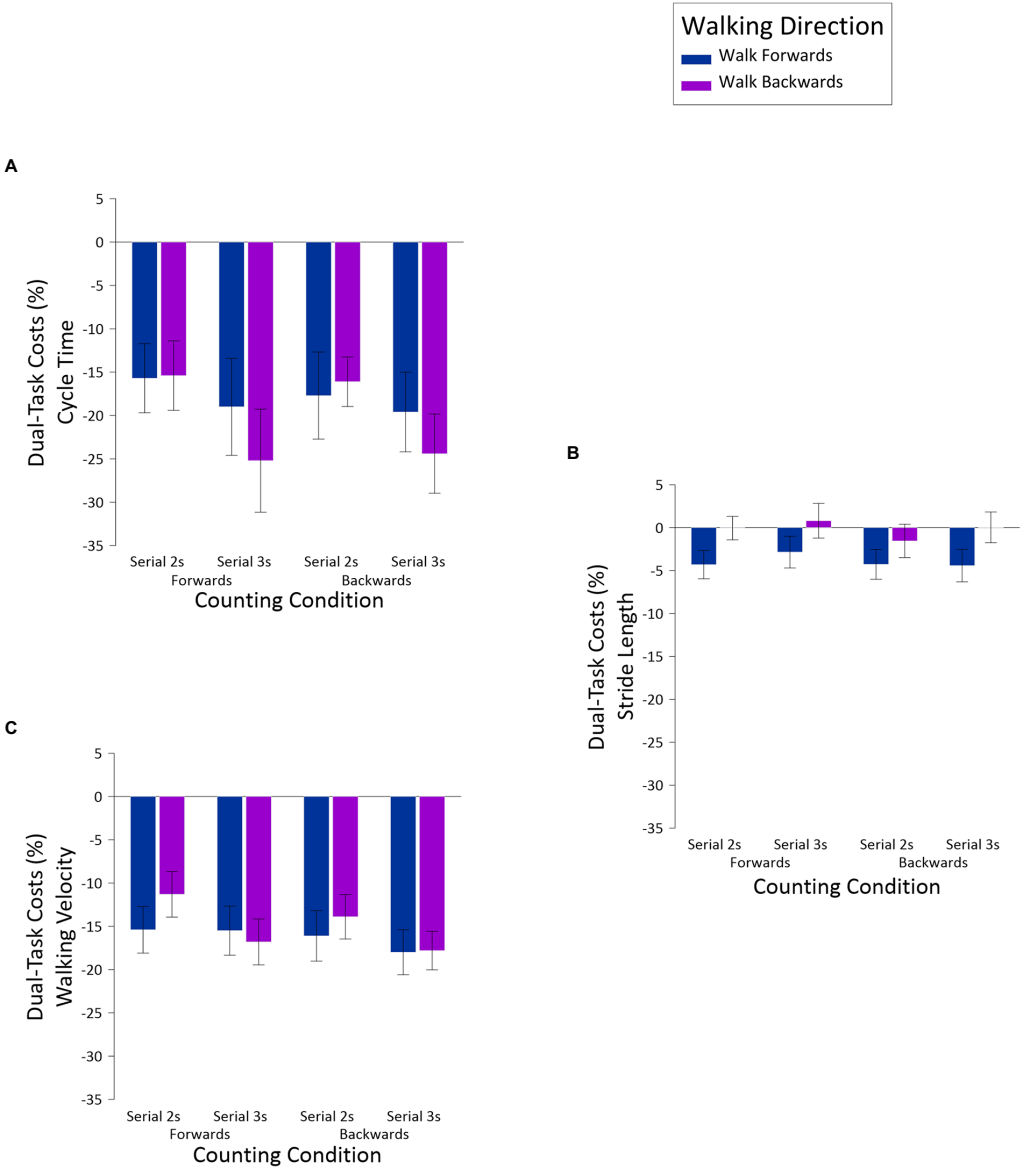
Dual-task costs for cycle time **(A)**, stride length **(B)**, and walking velocity **(C)**, as a function of *Walking Direction, Counting Direction* and *Counting Difficulty*.

Dual-task costs were analyzed in three-way ANOVAs, with the same factors of *Walking Direction*, *Counting Direction*, and *Counting Difficulty.* To recap, dual-task costs were calculated by taking the difference between single-task and dual-task conditions, divided by the single-task condition, and multiplied by 100 to create percentages. The results of these analyses appear in Figure 2. For cycle times (Figure 2A), there was a main effect for *Counting Difficulty*, *F*(1,23) = 8.38, *MSE* = 197.95, *p* = 0.008, *np^2^* = 0.27, with serial 2 s producing less of a dual-task cost (*M* = −16.22, *SE* = 3.55) than serial 3 s (*M* = −22.06, *SE* = 4.91). The only other significant result was the *Walking Direction* x *Counting Difficulty* interaction, *F*(1,23) = 4.39, *MSE* = 113.29, *p* = 0.047, *np^2^* = 0.16. Subsequent comparisons revealed that, when walking forward, the dual-task costs associated with serial 2 s (*M* = −16.69, *SE* = 4.42) versus serials 3 s (*M* = −19.31, *SE* = 4.86) did not differ, *t*(23) = 1.29, *ns*, but when walking backward, the costs associated with serial 2 s (*M* = −15.76, *SE* = 3.13) versus serial 3 s (*M* = −28.81, *SE* = 5.15), did differ, *t*(23) = 3.07, *p* = 0.005.

For stride lengths (Figure 2B), the only significant result was a main effect for *Walking Direction, F*(1,23) = 5.36, *MSE* = 127.37, *p* = 0.03, *np^2^* = 0.19, with walking forward producing more of a dual-task cost (*M* = −3.96, *SE* = 1.70) than walking backward (*M* = −0.19, *SE* = 1.43). As with the previous analyses for stride lengths, although the main effect of *Counting Direction* was not significant, *F*(1,23) = 3.70, *MSE* = 11.76, *p* = 0.067, *np^2^* = 0.14, there was a notable trend toward smaller dual-task costs when counting forward (*M* = −1.60, *SE* = 1.36) than counting backward (*M* = −2.55, *SE* = 1.38). Given that none of the remaining effects were significant, and that the results that were significant were very small,^3^ what is most notable about this analysis is that dual-task performance had a rather limited impact on stride lengths. Finally, for walking velocities (Figure 2C), the only significant result was a main effect of *Counting Difficulty*, *F*(1,23) = 7.28, *MSE* = 54.01, *p* = 0.013, *np^2^* = 0.24, with less of a dual-task cost for serial 2 s (*M* = −14.17, *SE* = 2.20), than serial 3 s (*M* = −17.03, *SE* = 2.28). Thus, and as predicted, a more challenging counting regime had a more globally deleterious impact on walking than a less challenging counting task.

#### Cognitive performance

**Figure 3:**
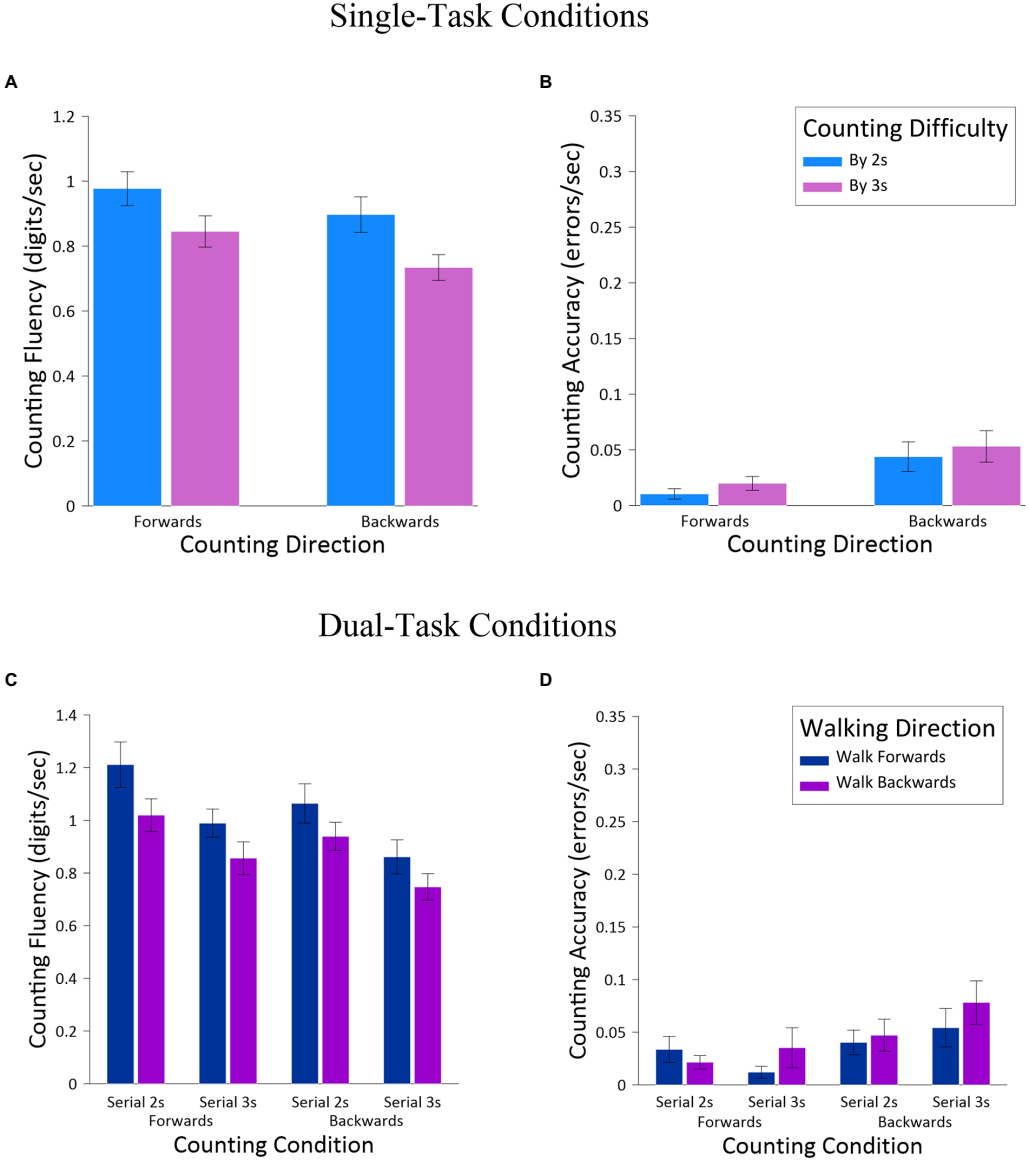
Single-task cognitive measures [counting fluency (**A**) and counting accuracy (**B**)] and dual-task measures [counting fluency (**C**) and counting accuracy (**D**)], as a function *Walking Direction, Counting Direction*, and *Counting Difficulty*.

For the single-task conditions, counting fluency and counting accuracy were analyzed in two-way ANOVAs, with the factors of *Counting Direction* and *Counting Difficulty*. The ANOVA for counting fluency (Figure 3A) produced main effects for *Counting Direction*, *F*(1,23) = 22.05, *MSE* = 0.010, *p* <. 001, *np^2^* = 0.49, and for *Counting Difficulty*, *F*(1,23) = 38.48, *MSE* = 0.013, *p* < 0.001, *np^2^* = 0.63, but no interaction between the factors, *F*(1,23) = 0.56, *MSE* = 0.010, *ns.* The effect of *Counting Direction* revealed greater fluency when counting forward (*M* = 0.91, *SE* = 0.05) than counting backward (*M* = 0.82, *SE* = 0.04), and the effect of *Counting Difficulty* revealed greater counting fluency for serial 2 s (*M* = 0.94, *SE* = 0.05) than serial 3 s (*M* = 0.79, *SE* = 0.04). The ANOVA for counting accuracy (Figure 3B) produced only a main effect for *Counting Difficulty*, *F*(1,23) = 7.28, *MSE* = 0.004, *np^2^* = 0.24, with fewer errors for serial 2 s (*M* = 0.02, *SE* = 0.004) than serial 3 s (*M* = 0.05, *SE* = 0.013).

For the dual-task conditions, counting fluency and counting accuracy were analyzed in three-way ANOVAs, with the same factors of *Walking Direction*, *Counting Direction,* and *Counting Difficulty* as already employed. For counting fluency (Figure 3C), this analysis produced only main effects, including differences for *Walking Direction*, *F*(1,23) = 20.44, *MSE* = 0.05, *p* < 0.001, *np^2^* = 0.47, *Counting Direction*, *F*(1,23) = 25.55, *MSE* = 0.02, *p* < 0.001, *np^2^* = 0.53, and for *Counting Difficulty*, *F*(1,23) = 40.36, *MSE* = 0.05, *p* < 0.001, *np^2^* = 0.64. These effects revealed greater fluency when walking forward (*M* = 1.03, *SE* = 0.07) than walking backward (*M* = 0.89, *SE* = 0.05), greater fluency when counting forward (*M* = 1.02, *SE* = 0.06) than counting backward (*M* = 0.90, *SE* = 0.06), and greater fluency for serial 2 s (*M* = 1.06, *SE* = 0.06) than serial 3 s (*M* = 0.86, *SE* = 0.06). For counting accuracy (Figure 3D) this analysis revealed a significant main effect only for *Counting Difficulty*, *F*(1,23) = 7.22, *MSE* = 0.006, *p* = 0.013, *np^2^* = 0.24, with fewer errors when serial 2 s (*M* = 0.03, *SE* = 0.01) than serial 3 s (*M* = 0.06, *SE* = 0.01). There was also a significant two-way interaction between *Counting Direction* and *Counting Difficulty*, *F*(1,23) = 4.66, *MSE* = 0.002, *p* = 0.042, *np^2^* = 0.17. Post-hoc comparisons (Tukey corrections) revealed that this interaction was due to a trend toward fewer errors when counting backward for serial 2 s (*M* = 0.02, *SE* = 0.006) than when counting backward for serial 3 s (*M* = 0.05, *SE* = 0.014), *t*(23) = 2.69, *p* = 0.059. In contrast, there was no difference in counting forward for serial 2 s (*M* = 0.01, *SE* = 0.005) than serial 3 s (*M* = 0.04, *SE* = 0.013), *t*(23) = 2.03, *ns*, although there was a notable trend toward a difference before correcting for multiple comparisons (*p* = 0.054).

**Figure 4:**
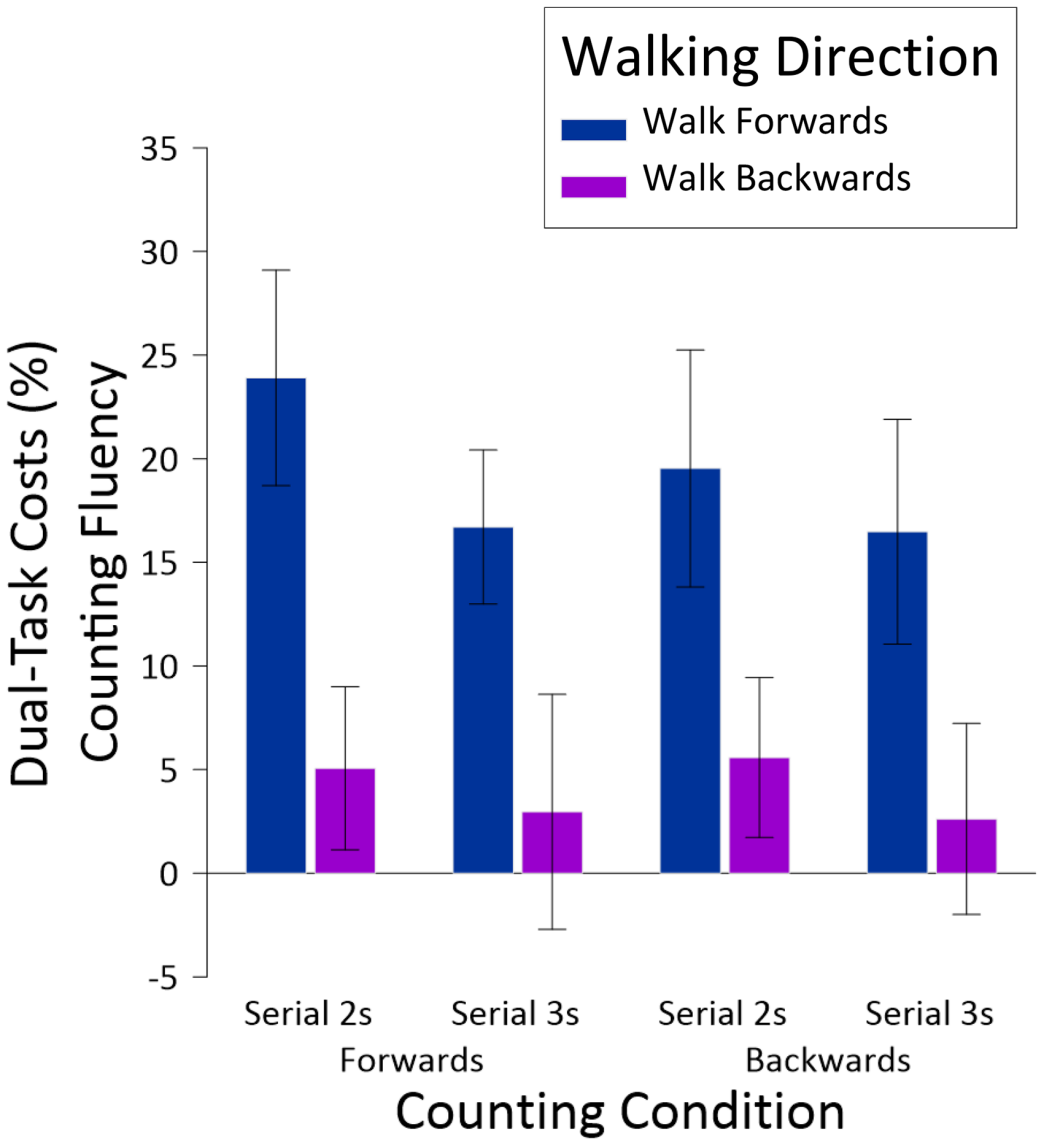
Dual-task costs for counting fluency, as a function *Walking Direction, Counting Direction*, and *Counting Difficulty*. Dual-task costs for counting accuracy could not be calculated due to the relative paucity of errors in the single-task condition (see figure 3).

Finally, the dual-task costs for counting fluency were analyzed. Given the low error rate in the single-task conditions (multiple participants had error rates of 0 for the various counting conditions), it was not possible to calculate dual-task costs for counting accuracy given that this calculation requires dividing the difference between single- and dual-task conditions by single-task performance. Dual-task costs for counting fluency were analyzed in a three-way ANOVA, with the same factors of *Walking Direction*, *Counting Direction*, and *Counting Difficulty*, and appear in Figure 4. The only result to emerge from this analysis was a main effect of *Walking Direction*, *F*(1,23) = 30.44, *MSE* = 359.20, *p* < 0.001, *np^2^* = 0.57, with significantly lower (more positive) dual costs associated with walking forward (*M* = 19.15, *SE* = 3.67) than walking backward (*M* = 4.05, *SE* = 3.14). What is most striking about this result, however, is that compared to counting by itself (single-task conditions), walking boosted counting fluency and thus represent negative dual-task costs, in other words dual-task benefits.

#### Correlations between motor performance and cognitive performance

A final series of analyses correlated motor and cognitive performance across participants for both the single-task and dual-task conditions. For single-task conditions, all of the measures were intercorrelated, although the cognitive measures were restricted to only counting fluency. These correlations appear in Table 1. Not surprisingly, the various kinematic variables were generally strongly intercorrelated, with the exception of relations between cycle times and stride lengths. Similarly, counting fluency values in the various cognitive conditions were also strongly intercorrelated. Most importantly, however, were the lack of relations between motor behavior and cognitive behavior when cognitive and motor tasks were performed independently. This lack of an effect is both anticipated and important. For the former, there is no reason why walking behavior in isolation should be in any way related to counting fluency in isolation. And for the latter, it indicates that any observed relations between dual-task motor and cognitive behaviors are not due to underlying relations between the single-task motor and cognitive components themselves.

**Table 1:** Inter-correlation matrix between motor performance (cycle time, stride length, walking velocity) and cognitive performance (counting fluency) variables in the single-task conditions.

			Cycle Time		Stride Length		Walk Velocity		Counting Fluency		
			WF	WB	WF	WB	WF	WB	CF		CB
									Serial 2 s	Serial 3 s	Serial 2 s
Cycle Time	WB		**0.750** ^****^								
Stride Length	WF		−0.341	−0.227							
	WB		0.044	0.080	**0.430** ^*^						
Walk Velocity	WF		**−0.837** ^***^	**−0.608** ^***^	**0.791** ^****^	0.195					
	WB		**−0.504** ^*^	**−0.635** ^***^	**0.505** ^*^	**0.705** ^****^	**0.604** ^***^				
Counting Fluency	CF	Serial 2 s	−0.236	−0.246	0.259	0.239	0.313	*0.374* *^C^*			
	CB	Serial 3 s	−0.067	−0.018	0.146	*0.358* *^D^*	0.136	0.302	**0.835** ^****^		
	CF	Serial 2 s	−0.289	−0.238	0.212	0.229	0.331	*0.377* *^B^*	**0.850** ^****^	**0.823** ^****^	
	CB	Serial 3 s	−0.218	−0.241	0.108	0.249	0.230	*0.404* *^A^*	**0.802** ^****^	**0.849** ^****^	**0.757** ^****^

* p < 0.05;

*** p < 0.005;

**** p < 0.0001;

A p ≤ 0.06;

B p ≤ 0.07;

C p ≤ 0.08;

D p ≤ 0.09.

Significant correlations are shown in **bold**; marginally significant correlations are shown in italics. WF: walk forward; WB: walk backward; CF: count forward; CB: count backward.

For the dual-task conditions and the dual-task costs, kinematic measures were correlated with the counting fluency data in each condition. These correlations appear in Table 2 and also present a reasonably clear pattern. For the dual-task measures, there were significant positive correlations between changes in stride length and counting fluency across all conditions, although the correlation in the walking forward, counting forward, serial 2 s condition was only marginally significant. These correlations indicate that when coordinating motor and cognitive behavior, participants who maintained optimum motor performance, at least in terms of stride lengths, were also those participants who maintained optimum cognitive performance.

**Table 2:** Correlations between motor performance variables (cycle time, stride length, and walking velocity) and cognitive performance (counting fluency) for the dual-task conditions, and the dual-task costs.

			Dual-task performance			Dual-task costs		
Dual-task condition			Cycle Time	Stride Length	Walking Velocity	Cycle Time	Stride Length	Walking Velocity
Walk Forward	Count Forward	Serial 2 s	0.033	0.380^A^	0.158	−0.340	0.076	−0.230
		Serial 3 s	0.016	0.550^***^	0.212	−0.118	0.112	−0.053
	Count Backward	Serial 2 s	0.059	0.572^***^	0.220	−0.064	0.360	0.119
		Serial 3 s	0.236	0.447^*^	0.072	−0.263	0.254	−0.117
Walk Backward	Count Forward	Serial 2 s	−049	0.422^*^	0.237	−0.354^B^	0.422^*^	−0.186
		Serial 3 s	0.104	0.457^*^	0.119	−0.069	−0.258	−0.294
	Count Backward	Serial 2 s	0.146	0.536^**^	0.288	−0.583^***^	0.470^*^	−0.115
		Serial 3 s	0.233	0.486^*^	0.074	−0.292	0.468^*^	−0.049

* p < 0.05;

** p < 0.01;

*** p < 0.005;

A p ≤ 0.07;

B p ≤ 0.0.

This interpretation is supported by the correlations for the dual-task costs, also shown in Table 2, although the patterns were more variable than those for the dual-task measures themselves. Still, there are again a relatively consistent set of positive correlations between the dual-task costs for stride length, and the dual-task costs for counting fluency, with the important caveat that this pattern was limited to (three of the four) walking backward conditions. As discussed previously, the negative dual-task costs associated with the fluency data indicate an actual benefit to performance in dual-task, versus single-task, conditions. As such, the positive correlations between dual-task costs in motor and cognitive performance indicate that those participants who experienced more detriment in motor performance stemming from the dual-task conditions were also those participants who received less facilitation to counting fluency from the dual-task demands.

#### Practice effects on single-task performance

Finally, it is of interest to explore the impact of potential practice effects in the single-task conditions. The decision to randomly order the single- and dual-task conditions, although solid experimental design, does produce the possibility of varying performance as a function of prior practice and experience. Specifically, it is possible that single-task performance might vary systematically depending on whether it occurred earlier or later in the experimental session. Later occurrence could improve performance due to participants having received practice with this task in a previous dual-task condition. Given that the single-task conditions provide a baseline for dual-task costs, it is of interest to explore this. possibility. To examine this idea, the various dependent measures in the single-task conditions were examined as a function of when they occurred in the experimental protocol. Thus, motor measures (cycle time, stride length, walking velocity) in the two single-task conditions (walk forward, walk backward) were correlated with the block number for when these conditions occurred in the experimental session. Similarly, cognitive measures (counting fluency only, given the ceiling effects in counting accuracy) in the four single-task conditions (count forward by 2 s, count forward by 3 s, count backward by 2 s, count backward by 3 s) were correlated with the block number for when these conditions occurred.

Of these 10 correlations (six motor, four cognitive) two were significant – the correlation for walking velocity in the walk backward condition, *r*(22) = 0.447, *p* = 0.028, and for counting backward by 3 s, *r*(22) = 0.502, *p* = 0.012. In both cases, the direction of this correlation indicates improved performance (greater velocity, increased fluency) associated with later occurrence in the experimental session, suggestive of some potential impact of prior experience from a previously occurring dual-task condition.

Two points should be noted regarding these analyses. First, the fact that only two of these 10 correlations were significant, with only one each for the motor and cognitive variables is, at best, weak evidence for an effect for previous practice in this dual-task paradigm. Moreover, in both cases, the significance of these correlations does not survive corrections for multiple comparisons. Thus, the overall impact of this practice is debatable, and potentially a statistical aberration. Second, and in contrast to the first point, it is at least noteworthy that the two effects that did show some level of effect both occurred in the most difficult conditions for both motor and cognitive tasks. Together, although there is no definitive evidence for practice effects in this study, the idea of investigating the impact of motor and cognitive practice on dual-task performance in this context is nevertheless intriguing. Indeed, such an investigation would fit nicely into the existing body of literature exploring how practice influences dual-task behaviors (Strobach and Schubert, 2016; Strobach, 2020; Huo et al., 2022).

## Discussion

To summarize the principal results of this study, this experiment examined the impact of simultaneous manipulations of the difficulty of concurrent motor and cognitive tasks. For motor variables, this study found that, although manipulations of difficulty did modify motor performance, both when performed separately (single-task conditions) and concurrently (dual-task conditions), the principal cost to behavior when engaging in dual-task conditions, as indicated by the dual-task costs, was more consistently driven by cognitive manipulations, and only sporadically by motor manipulations. For cognitive variables, although these manipulations of difficulty again varied performance in isolation (single-task) and in combination (dual-task), the principle cost to behavior was driven by motor manipulations. Most dramatically, dual-task requirements actually enhanced performance relative to single-task conditions, producing dual-task benefits. Finally, motor and cognitive performance within each of the various dual-task conditions were correlated, such that participants who showed the most impact on motor performance also showed the most impact on cognitive performance.

Focusing on the motor variables, one of the more interesting findings was that, although gait parameters were influenced by the dual-task manipulations, by and large dual-task costs were observed only for temporal (cycle time) and spatiotemporal (walking velocity) parameters, but not for the spatial (stride length) variable. Thus, the demands of simultaneous motor and cognitive conditions caused participants to slow their walking overall, but not to modify the distance of their strides. Interestingly, having experimental manipulations impact primarily on temporal, but not spatial, parameters of walking has been reported by other researchers. Specifically, work on auditory–motor entrainment in walking (Thaut, 2008, 2015), in which researchers examine the impact on gait of different forms of auditory information (i.e., metronome beats at different tempos) have generally found that such input has its most robust impact on temporal and spatiotemporal variables of gait, with less consistent effects on spatial properties (Lim et al., 2005; Willems et al., 2006; Rochester et al., 2007).^4^ Such an effect is understandable when considered from a biomechanical vantage point, in which the principle determinants of stride length in bipedal locomotion derives from pelvic width, and the degree of flexion and extension at the hips leading to grater pelvic rotation (Rak, 1991; Gruss et al., 2017). Because the current manipulations do not place significant demands on, or require modification of the fundamental biomechanics of walking (indeed, backward walking has often been characterized as requiring comparable biomechanics to forward walking, Winter et al., 1989; Grasso et al., 1998; van Deursen et al., 1998), it is thus not surprising that little impact was observed on stride lengths.

A second intriguing, and novel effect arising from the motor performance measures is that the principal dual-task cost observed on these parameters was driven by cognitive manipulations, and not by motor manipulations. Thus, although the motor manipulation in this study influenced motor behavior overall, this difference did not increase when combined with a simultaneous cognitive task. This finding suggests that the cognitive resources required to accomplish the motor task were, surprisingly, not undermined by the increased resources required for a simultaneous cognitive task. Thus, motor performance proceeded apace depending on motor demands, irrespective of the demands of the cognitive task.

Theoretically, this result argues against central-resource models of attention and performance (Kahneman, 1973; Neumann, 1996; Wickens, 2008) given that one of the primary characteristics of such models is that attention is ultimately of limited capacity. Thus, resources focused on one task should, by definition, detract from the resources available for another task. Instead, these findings are more aligned with the multiple resource account (Navon and Gopher, 1979; Wickens, 2002, 2008, 2021) that posits the existence of multiple attentional mechanisms, each with its own limited resources that are aligned to a given aspect of performance.

Although intriguing, ultimately these findings are suggestive at best, given that it is difficult to fully disentangle predictions from these two accounts within the present context. One could, for instance, support a central resource model by positing that the resources required by the motor task were essentially negligible, and thus failed to significantly detract from the resources available overall. Although straining credulity with regards to backward walking, given the highly practiced, automatic nature of walking for adults, this explanation is reasonable on some level. Interestingly, such an explanation does then predict that if one were to employ a more difficult walking task, such as obstacle avoidance, barrier clearance, or gap crossing, or if one were to examine participants for whom walking is not automatic, and more resource laden, such as young children, the dual-task costs of walking manipulations might be more prevalent.

Turning to the cognitive performance, probably the most intriguing finding involves the occurrence of negative dual-task costs for counting fluency, with participants’ counting fluency actually increasing when performing a dual-task involving forward and backward walking, relative to counting fluency when stationary. Interestingly, such paradoxical negative dual-task costs, or more accurately dual-task benefits, have been found (occasionally) by other researchers, with studies demonstrating improved performance in dual-task conditions, relative to single-task conditions (Beurskens et al., 2014; Yu and Huang, 2017). As an example, Beurskens et al. (2014) found that, with younger adults, simultaneous performance of a walking and verbal memory task produced improved walking performance (in terms of step length, step duration, and the number of steps), relative to single-task conditions. One important observation is that the studies that have demonstrated such dual-task benefits have typically observed such effects on motor performance, and not on cognitive behavior. Accordingly, the occurrence of dual-task benefits for cognitive processing is a significantly novel finding of the current project, particularly given that participants were told to consider motor task as the primary task.

Of course, demonstrating this effect raises the obvious question of the mechanism underlying why dual-task conditions increased counting fluency in this study. One possible explanation stems from the potential for auditory–motor entrainment in the current task (Thaut, 2008, 2015). Over the years researchers have recognized that auditory input can entrain motor behavior, such that a rhythmic auditory stimulus has a positive stabilizing effect on gait (Spaulding et al., 2013; Yoo and Kim, 2016; Ghai et al., 2018). Interestingly, this entrainment has generally been seen as unidirectional, with auditory rhythms influencing gait production, but not vice versa. However, entrainment simply assumes that the frequency of one (physical or biological) system influences the frequency of another system; as such, there is no reason why such effects should not be bidirectional, with motor rhythmicity influencing auditory production. In this vein, the present results could be explained through the rhythm of the walking tasks entraining the rhythm of counting, varying fluency as a function of walking cadence. In keeping with this hypothesis, the fact that the dual-task costs for counting fluency were strongly influenced by walking direction supports this entrainment hypothesis. Given that one of the principal impacts of walking direction was to modify temporal parameters of gait, if auditory production was entrained to walking cadence then one would anticipate seeing a strong relation between counting fluency and parameters explicitly affecting temporal gait cycle.

Of course, although the current data are consistent with this explanation, this experiment was not designed to test auditory–motor entrainment in counting. Specifically, to demonstrate such an effect one would look for an impact of explicit manipulations of walking speed on counting fluency, whether the frequency content of stepping and counting time series are related (i.e., cross-spectral analyses), whether auditory and motor behavior explicitly synchronizes, and so on. Such data would more thoroughly address the possibility of auditory counting entrainment based on motor behavior. Accordingly, this explanation highlights an exciting future direction for research.

As an alternative explanation, it is possible that the observed dual-task benefit may be accounted for by general increased vigilance in participants when walking compared to when they were stationary. If this were true, however, one would predict that *both* counting fluency and counting accuracy would increase in dual-task conditions (i.e., show dual-task benefits), relative to the single-task conditions. In contrast to this prediction, although the counting fluency did increase during the dual-task condition, the counting accuracy did not show dual-task costs. Accordingly, a general increase in arousal/vigilance cannot account for this novel finding.

Another noteworthy result arising from the counting data involves the consistently observed difference in the impact of counting direction and counting difficulty manipulations. Although both factors influenced single-task performance, in a dual-task context counting difficulty consistently effected motor behavior, whereas counting direction produced only sporadic effects. Although the former result is understandable, the latter finding is surprising, and somewhat at odds with the existing literature. Both manipulations were chosen because of their documented effects on cognitive processing, and the findings of the single-task conditions did confirm that both factors do exert such an influence on processing.

One possible explanation for the inconsistent effects in dual-task context is that, similar to the previously discussed lack of a dual-task cost for walking manipulations, it could simply be that addition and subtraction are so highly automatized that they fail to differentially draw on resources in a dual-task situation. In support of this explanation, it is worth noting that the majority of previously discussed literature pertaining to processing differences between addition and subtraction is developmental in nature. Converging with the previous discussion, investigation of these manipulations with child participants appears to be especially relevant. This population would likely show a more dramatic effect of this variable.

Finally, this study also demonstrated that the impact of dual-task manipulations on participants is correlated within individual participants, with participants showing internal consistency in their respective abilities to integrate motor and cognitive tasks. Surprisingly, there exists little work about individual differences in dual-task performance, although there are some notable exceptions in work examining dual-task abilities in children and as a function of aging in the elderly (Verhaeghen et al., 2003; Wollesen and Voelcker-Rehage, 2014; Brustio et al., 2017), and in some studies with adults (Brüning and Manzey, 2018; Brüning et al., 2020), please see a recent review and synthesis by Broeker et al. (2022). And for the handful of studies that have investigated this question, the focus has been on questions such as whether performance on individual task components predicts performance in a multicomponent test (Lansman and Hunt, 1981, 1982), the role of cognitive style on dual-task performance (Nishizaki and Osaka, 2006), and whether differences in participants’ response strategies predicts dual-task performance (Damos and Smist, 1980; Damos et al., 1983; Damos, 1984). Surprisingly, in our review of the literature we have been unable to find any work that directly addresses whether the impact of dual-task requirements affects both components of a dual-task comparably within individual participants. Thus, this result is (to our knowledge) the first finding of its kind, a fact that likely arises because, as highlighted in the introduction, researchers typically do not simultaneously manipulate the difficulty of multiple components of a dual-task, and when they do, they neglect to assess and compare performance in both tasks.

On some level, it is not surprising that the dual-task manipulations comparably influenced motor and cognitive processing with individual participants. Certainly individual differences factor into attentional resources and general processing capabilities (Hunt et al., 1989; Conway et al., 2021; Mashburn et al., 2021; Unsworth et al., 2021), and as such, finding that the allocation of such resources to the multiple tasks in a dual-task context is correlated within individuals is not surprising. On a different level, though, the fact that motor and cognitive performance in dual-task conditions was related is unusual given that participants were instructed to focus on one task (walking), and not equally on the two behaviors. Such instructions should have, at the least, severely weakened such a relation between motor and cognitive performance. That said, given the novelty of this result, more research is required before one can fully make sense of the implications of correlated motor and cognitive performance under dual-task conditions.

In conclusion, the current study has highlighted an array of novel results pertaining to the integration of motor and cognitive behaviors when both dimensions are simultaneously manipulated in their respective demands and levels of difficulty. These findings have also highlighted multiple lines of future investigation that would be profitably explored in future research. Ultimately, this work sheds light on the complex processes involved in performance of action in the real world, which requires attention to and the integration of multiple sources of information, along with the production of a host of simultaneous motor and cognitive behaviors.

## Data availability statement

The raw data supporting the conclusions of this article will be made available by the authors, without undue reservation.

## Ethics statement

The studies involving human participants were reviewed and approved by Social Sciences, Humanities & Education REB of the University of Toronto. The patients/participants provided their written informed consent to participate in this study.

## Author contributions

AM and TC were primarily responsible for the experimental design and wholly responsible for data collection. Am and MS were responsible for data analysis and initial manuscript preparation. All authors contributed to the article and approved the submitted version.

## Funding

The current research was funded by NSERC Discovery Grant awarded to MS.

## Conflict of interest

The authors declare that the research was conducted in the absence of any commercial or financial relationships that could be construed as a potential conflict of interest.

## Publisher’s note

All claims expressed in this article are solely those of the authors and do not necessarily represent those of their affiliated organizations, or those of the publisher, the editors and the reviewers. Any product that may be evaluated in this article, or claim that may be made by its manufacturer, is not guaranteed or endorsed by the publisher.

## Footnotes

^1^The sample size was based on an *a priori* power analysis using G*Power 3.1 (Faul et al., 2009), focusing on the *Walking Direction × Counting Direction × Counting Difficulty* interaction. The following values were used to determine an appropriate sample size: effect size of *f* = 0.4, α error probability = 0.05, power (1-β probability) = 0.95, number of groups = 3, number of measurements = 2, correlation among repeated-measures = 0.5, nonsphericity correction ɛ = 1. Based on these values, a sample size of 24 participants was suggested.

^2^It is important to note that the term ‘severe’ was not used strictly with regards to the clinical definition of a ‘severe head injury,’ but rather to help participants distinguish between simple head bumps and more serious head injuries, such as concussions. One participant did, in fact, report having experienced a severe head injury. However, this participant further reported that their concussion occurred over ten years prior to data collection. Moreover, the data for this participant did not deviate significantly from the rest of the participants. Therefore, this participant’s data was included in the final analysis.

^3^Given that dual-task costs are standardized percentage measures (difference between single- and dual-task conditions, divided by the single-task condition), the values for these costs regardless of the underlying scale of the measure are comparable across measures. Thus, a dual-task cost of 30% for a particular manipulation represents a significantly larger impact than a dual-task cost of 5% for a second measure, regardless of which measures are being compared.

^4^Schmuckler, M. A., and Paolozza, A. (submitted). Auditory influences on walking: children’s walking to the beat. Dev. Psych.
